# Circulating Pro-Resolving Mediators Maresin-1 and Maresin-2 and Adipolin/CTRP12 in Adults with Atopic Dermatitis: A Case–Control Study

**DOI:** 10.3390/medicina62101923

**Published:** 2026-10-06

**Authors:** Mahir Dığış, Kısmet Kaya, Betül Demir, Zeynep Hilal Gündüz, Kadir Ceren, Ahmet Furkan Süner, Süleyman Aydın

**Affiliations:** 1 Department of Dermatology and Venereology, Faculty of Medicine, Fırat University, 23119 Elazığ, Türkiye; 2 Department of Public Health, Faculty of Medicine, Düzce University, 81620 Düzce, Türkiye; 3 Department of Medical Biochemistry, Faculty of Medicine, Fırat University, 23119 Elazığ, Türkiye

**Keywords:** atopic dermatitis, maresin-1, maresin-2, adipolin, CTRP12, specialised pro-resolving mediators, lactate dehydrogenase

## Abstract

*Background and Objectives:* Defective resolution of inflammation may contribute to chronicity in atopic dermatitis (AD). Maresin-1 (MaR1) and Maresin-2 (MaR2) are docosahexaenoic acid-derived specialised pro-resolving mediators, and adipolin/C1q/TNF-related protein 12 (CTRP12) is an adipokine with anti-inflammatory properties; to our knowledge, none has been evaluated in the circulation of patients with AD. *Materials and Methods:* In this single-centre case–control study, 44 adults with AD and 44 healthy controls were enrolled. Serum MaR1, MaR2, and adipolin were measured by enzyme-linked immunosorbent assay, and disease severity was graded with the Scoring Atopic Dermatitis (SCORAD) index and the Eczema Area and Severity Index (EASI). Between-group comparisons were complemented by covariate-adjusted models (age, sex, body mass index, smoking), Spearman correlation, receiver operating characteristic (ROC) analysis, and false discovery rate control. *Results:* Serum adipolin (3.16 vs. 3.23 ng/mL; *p* = 0.211), MaR1 (140.31 vs. 141.09 ng/L; *p* = 0.815), and MaR2 (67.56 vs. 72.29 pg/mL; *p* = 0.512) did not differ between patients and controls, and the null findings persisted after adjustment (all *p* ≥ 0.306). None of the three biomarkers correlated with SCORAD or EASI (all *p* ≥ 0.389) or discriminated patients from controls (area under the ROC curve 0.459–0.578). Results were unchanged when measurements outside the assay calibration ranges (adipolin, 67.0% below; MaR1, 14.8% and MaR2, 13.6% above) were censored. Serum lactate dehydrogenase was higher in patients (217.52 vs. 183.39 U/L; q = 0.004) and correlated with SCORAD (ρ = 0.404) and EASI (ρ = 0.349). Adipolin and MaR1 were strongly correlated (ρ = 0.815), including among samples with adipolin below the calibration range (ρ = 0.640). *Conclusions:* Circulating MaR1, MaR2, and adipolin/CTRP12 were not altered in adults with AD and do not appear useful as standalone diagnostic or severity biomarkers, although compartmentalised cutaneous activity cannot be excluded. The adipolin–MaR1 correlation showed features of an analytical artefact and should not be interpreted biologically.

## 1. Introduction

Atopic dermatitis (AD) is a chronic, relapsing inflammatory skin disease that arises from epidermal barrier dysfunction and immune dysregulation. Acute lesions are driven mainly by Th2/Th22 pathways, whereas chronic disease shows broader immune activation that also involves Th1 and Th17 responses [1,2]. Beyond excessive inflammatory activity, inadequate resolution of inflammation is increasingly considered a contributor to disease persistence: failure to resolve inflammation may prevent the return to tissue homeostasis and sustain chronic or recurrent skin inflammation in AD [3,4].

The resolution of inflammation is not a passive consequence of waning inflammatory signals but an actively regulated process mediated by specialised pro-resolving mediators (SPMs) such as resolvins, protectins, lipoxins, and maresins [3,4]. These lipid mediators, derived mainly from polyunsaturated fatty acids, regulate leukocyte trafficking, macrophage polarisation, phagocytosis, efferocytosis, and the restoration of tissue homeostasis [4,5]. AD has been linked to reduced omega-3-derived SPMs and to a shift from pro-resolving towards pro-inflammatory lipid mediators, although direct human evidence remains scarce [3,4]. Experimental studies further suggest that SPMs attenuate AD-relevant pathways by reducing IL-4, IL-5, IL-13, and IL-17A responses, inhibiting immunoglobulin E (IgE) production, enhancing regulatory T-cell function and M2 macrophage polarisation, and preserving epidermal barrier integrity [4].

Maresins are SPMs derived from docosahexaenoic acid (DHA) and produced mainly by macrophages during the resolution of inflammation. Maresin-1 (MaR1), the first member to be identified, is a potent anti-inflammatory and pro-resolving mediator that limits neutrophil recruitment and promotes macrophage phagocytosis and efferocytosis [5]. In an experimental model of skin inflammation, MaR1 reduced IL-23 receptor expression, IL-17A production, and inflammatory cell recruitment and attenuated skin inflammation [6]. MaR1 also reduced UVB-induced skin oedema, neutrophil accumulation, keratinocyte apoptosis, and epidermal thickening [7]. Furthermore, the effects of MaR1 on epithelial tight-junction proteins such as claudin-1 and ZO-1 suggest a possible mechanistic link to the barrier dysfunction that is central to AD [8]. Despite these mechanistic data, clinical evidence on circulating MaR1 in AD is lacking; to our knowledge, no study has compared serum MaR1 concentrations between patients with AD and healthy individuals.

Maresin-2 (MaR2), a second DHA-derived macrophage mediator, reduces neutrophil recruitment and enhances macrophage phagocytosis, two key processes in the resolution of inflammation [9]. In a murine model of allergic airway inflammation, MaR2 decreased inflammatory cell infiltration, Th2 responses, oxidative stress, and NLRP3 inflammasome activation, pathways that overlap with those involved in AD [10]. Furthermore, MaR2 has been associated with tissue repair and the resolution of inflammation in experimental models of metabolic and mucosal diseases [11,12]. However, the evidence for MaR2 is largely preclinical, its receptor has not been fully characterised, and clinical studies of circulating MaR2 in human inflammatory diseases are scarce [8,13]. Whether systemic MaR2 concentrations are altered in AD is therefore unknown.

Alongside lipid-derived resolution mediators, adipokines have been proposed as links between metabolic homeostasis and cutaneous inflammation. Several adipokines, including adiponectin, leptin, resistin, visfatin, apelin, and zinc-α2-glycoprotein, are altered in AD, although the direction and magnitude of these changes differ between molecules and patient groups [14,15,16]. Adipolin, also known as C1q/TNF-related protein 12 (CTRP12), is an adipose-derived adipokine with insulin-sensitising and anti-inflammatory effects [17]. Experimental studies suggest that adipolin inhibits macrophage production of TNF-α, IL-6, and MCP-1 via TGF-βRII/Smad2 signalling and promotes polarisation towards an anti-inflammatory M2 phenotype through the miR-155-5p/LXRα pathway [18,19]. Circulating CTRP12 concentrations are reduced in coronary artery disease and correlate inversely with inflammatory cytokines and insulin resistance [20]. Nevertheless, despite the growing literature on other adipokines in AD, to our knowledge no clinical data are available on circulating adipolin/CTRP12 concentrations in AD.

Although maresins and adipolin originate from distinct biochemical systems, they converge functionally on macrophage biology: both CTRP12 and SPMs suppress macrophage-mediated inflammatory signalling and promote resolution-associated phenotypes. However, no direct molecular interaction between adipolin and maresin biosynthesis or signalling has been demonstrated [18,19,21]. Simultaneous assessment of these mediators may therefore provide complementary information on systemic anti-inflammatory and pro-resolving pathways in AD.

From a clinical perspective, AD still lacks a validated circulating biomarker for routine use. Diagnosis rests on clinical criteria, and severity is graded with physician-scored instruments such as the Scoring Atopic Dermatitis (SCORAD) index and the Eczema Area and Severity Index (EASI), which are subject to inter-observer variability. Among the many laboratory candidates investigated, serum thymus and activation-regulated chemokine (TARC/CCL17) has the strongest evidence for a correlation with disease severity, yet no circulating marker has gained widespread acceptance in routine practice [22,23]. An objective serum marker reflecting the resolution phase of inflammation could, in principle, complement clinical scoring, support the monitoring of disease activity, and help to identify patients in whom impaired resolution contributes to chronicity. At present, however, MaR1, MaR2, and adipolin can be quantified only with research-use-only immunoassays or, for the lipid mediators, with mass-spectrometry-based profiling; to our knowledge, no assay validated for in vitro diagnostic use is available, and these measurements are not part of routine clinical laboratory testing. Any consideration of clinical implementation or of cost-effectiveness therefore presupposes evidence that circulating concentrations differ between patients and healthy individuals or track disease severity. Establishing whether this prerequisite is met was the rationale for the present study.

The aim of this study was to compare serum concentrations of MaR1, MaR2, and adipolin/CTRP12 between adults with AD and healthy controls and to examine their relationships with clinical disease severity. We also assessed the associations among these mediators and their ability to discriminate patients with AD from healthy individuals.

## 2. Materials and Methods

### 2.1. Study Design and Setting

This single-centre case–control study was conducted at the Department of Dermatology and Venereology and the Department of Medical Biochemistry, Faculty of Medicine, Fırat University (Elazığ, Türkiye). Participants were sampled according to disease status (adults with AD as cases and healthy adults without AD as controls), and all clinical assessments and blood sampling were performed at a single study visit. The study was purely observational: no intervention was administered and no experimental manipulation of participants or samples was undertaken. Participants were enrolled consecutively between 10 July and 10 August 2026. No formal a priori sample size calculation was performed; the study used a convenience sample of all eligible individuals recruited during the enrolment period. The manuscript was prepared in accordance with the STROBE (Strengthening the Reporting of Observational Studies in Epidemiology) statement for observational studies.

### 2.2. Ethical Approval

The study protocol was approved at the session of the Non-Interventional Research Ethics Committee of Fırat University held on 9 July 2026 (session no. 2026/10-15; document no. 49659). All procedures were performed in accordance with the Declaration of Helsinki. Written informed consent was obtained from all participants before enrolment.

### 2.3. Participants

Eighty-eight participants were enrolled: 44 adult patients with AD attending the dermatology outpatient clinic and 44 healthy volunteers recruited during the same study period.

Patients were eligible if they were aged ≥18 years and had a diagnosis of AD established according to the Hanifin and Rajka criteria; histopathological confirmation was obtained where clinically indicated. Patients who had received systemic immunosuppressive, immunomodulatory, or biologic therapy or phototherapy within the preceding two months were excluded. In accordance with the study protocol, patients who had used any topical therapy for AD (topical corticosteroids, topical calcineurin inhibitors, or emollients) within the preceding two weeks, and patients taking oral antihistamines, were likewise excluded so that no patient was receiving systemic or topical treatment for AD at the time of blood sampling. Controls were healthy volunteers with no known comorbidity and no active dermatological, inflammatory, infectious, or autoimmune condition; all controls were screened for a personal history of atopic disease (atopic dermatitis, allergic rhinitis, or asthma) and those with a positive history were excluded. Exclusion criteria applied to both groups were pregnancy or lactation, diabetes mellitus, any malignancy, and any concomitant dermatological disorder other than atopic dermatitis.

Age, sex, height, body weight, body mass index (BMI), occupation, smoking status, disease duration, comorbidities, and current medications were recorded for all participants.

### 2.4. Assessment of Disease Severity

Disease severity was assessed in all patients using the SCORAD index and the EASI. All scoring was performed by the same dermatologist throughout the study period, eliminating inter-observer variability. SCORAD was categorised as mild (<25), moderate (25–50), or severe (>50) according to the European Task Force on Atopic Dermatitis convention [24], and EASI as mild (1.1–7.0), moderate (7.1–21.0), or severe (21.1–50.0) according to Leshem et al. [25]. Only the total SCORAD and EASI scores were recorded; the objective and subjective components of SCORAD were not available separately.

### 2.5. Blood Sampling and Routine Laboratory Analyses

After a minimum 8 h overnight fast, 5 mL of venous blood was drawn from the antecubital vein of each participant between 08:00 and 10:00 h in order to minimise the influence of diurnal variation. Samples were centrifuged at 3000 rpm for 10 min and the separated serum was aliquoted and stored at −80 °C until analysis; repeated freeze–thaw cycles were avoided.

Complete blood count, erythrocyte sedimentation rate, C-reactive protein, glycated haemoglobin (HbA1c), total IgE, and a routine biochemistry panel (glucose, lipid profile, hepatic and renal function tests, lactate dehydrogenase (LDH), thyroid-stimulating hormone) were measured on fresh samples using the automated analysers in routine clinical use at our institution.

### 2.6. Measurement of Serum MaR1, MaR2, and Adipolin

Serum concentrations were determined by commercial enzyme-linked immunosorbent assay (ELISA) kits according to the manufacturers’ instructions:

MaR1—YLBiont (Shanghai, China), cat. no. YLA4082Hu; detection range 0.5–300 ng/L; analytical sensitivity 0.19 ng/L; intra-assay coefficient of variation (CV) <8%, inter-assay CV <10%.

MaR2—BT-Lab (Shanghai, China), cat. no. EA0256Hu; detection range 1.56–100 pg/mL; analytical sensitivity 0.83 pg/mL; intra-assay CV <10%, inter-assay CV <12%.

Adipolin (CTRP12)—Sinogeneclon (Hangzhou, China), cat. no. SG-15346; detection range 1.25–22.5 ng/mL; analytical sensitivity 0.43 ng/mL; intra-assay CV <8%, inter-assay CV <10%.

Each analyte was measured on its own kit-specific microplates. Patient and control samples were co-assayed on the same plates for each analyte so that no group was confined to a separate plate and between-plate variation could not act as a systematic group-level effect. All samples were run in duplicate, and the mean of the two readings was used for analysis. Concentrations were derived from a standard curve prepared on each plate as specified by the manufacturer and are expressed in ng/L for MaR1, pg/mL for MaR2, and ng/mL for adipolin. For adipolin, serum was diluted 1:5 before assay as recommended by the manufacturer, and the measured concentrations were multiplied by the dilution factor; the effective calibration range for adipolin in undiluted serum was therefore 6.25–112.5 ng/mL. Serum was assayed undiluted for MaR1 and MaR2. Samples that read outside the calibration range were not re-assayed at a different dilution; their concentrations were obtained by extrapolation of the standard curve, and their numbers are reported in Section 3.4. Two adipolin observations in the control group were recorded as below the analytical detection limit and were coded as 0 for statistical analysis; there were no missing observations for the three primary biomarkers. Absorbance was read on a BioTek Epoch 2 microplate reader, and plates were washed with a Bio-Tek ELx50 microplate washer (both from Winooski, VT, USA). The coefficients of variation stated above are those reported by the manufacturers. The laboratory reported only the mean of each duplicate pair; the individual duplicate readings and the plate layouts were not available to the authors, so intra-assay coefficients of variation could not be calculated from our own data.

### 2.7. Outcomes

The pre-specified primary outcomes were the serum concentrations of MaR1, MaR2, and adipolin/CTRP12, compared between patients with AD and healthy controls. Secondary outcomes were (i) the correlation of each biomarker with disease severity (SCORAD and EASI) within the patient group; (ii) the correlations of the three biomarkers with one another and with total IgE; and (iii) the ability of each biomarker to discriminate patients from controls, expressed as the area under the receiver operating characteristic curve. Between-group comparisons of routine haematological and biochemical parameters were exploratory. The analyses relating serum LDH to disease severity and to the three biomarkers were not pre-specified; they were undertaken post hoc, after LDH emerged as the only routine parameter that differed between the groups following correction for multiple testing.

### 2.8. Statistical Analysis

Analyses were performed using R v4.2.3 (R Foundation for Statistical Computing, Vienna, Austria). Normality of continuous variables was assessed with the Shapiro–Wilk test together with visual inspection of histograms and quantile–quantile plots. Normally distributed variables are presented as mean ± standard deviation and compared between groups with the independent-samples t-test; non-normally distributed variables are presented as median (25th–75th percentile) and compared with the Mann–Whitney U test. Categorical variables are presented as frequency (percentage) and compared with Pearson’s chi-square test. For the nonparametric comparisons of the three primary biomarkers, rank-biserial correlation was used as an effect-size measure. Associations between continuous variables were assessed with Spearman’s rank correlation coefficient (ρ). To examine whether the primary between-group findings were robust to potential confounding, additional multivariable ordinary least-squares regression models were fitted separately for adipolin, MaR1, and MaR2, with study group as the main predictor and age, sex, BMI, and current smoking status entered as covariates. Because the biomarker distributions were markedly right-skewed and raw-scale model residuals did not satisfy normality adequately, concentrations were transformed as log(1+x) for these sensitivity models. Adjusted group coefficients (β) with 95% confidence intervals (CIs) are reported. The ability of serum adipolin, MaR1, and MaR2 to discriminate patients from controls was evaluated by receiver operating characteristic (ROC) analysis; areas under the curve (AUC) with 95% confidence intervals are reported, and the *p*-value accompanying each AUC tests the null hypothesis of no discrimination (AUC = 0.5). Serum adipolin, MaR1, and MaR2 were pre-specified as the primary outcomes. For the 41 exploratory routine laboratory comparisons (the 30 variables shown in Table 1 and 11 further variables reported in Table S1), the Benjamini–Hochberg procedure was used to control the false discovery rate; the same procedure was applied within the post hoc family of LDH correlation analyses (five tests: LDH versus SCORAD and EASI in patients, and LDH versus adipolin, MaR1, and MaR2 in the pooled cohort). Other exploratory correlation *p*-values were not adjusted for multiplicity. A two-sided *p*-value <0.05 was considered statistically significant; for Benjamini–Hochberg-adjusted analyses, q <0.05 was considered significant.

Because a proportion of measurements lay outside the calibration ranges of the assays (Section 2.6), post hoc sensitivity analyses were performed. First, MaR1 and MaR2 values above the upper calibration limit (300 ng/L and 100 pg/mL, respectively) and adipolin values below the effective lower calibration limit (6.25 ng/mL) were set to the respective limit, and the between-group comparisons, the correlations with SCORAD and EASI, and the ROC analyses were repeated. Second, the proportions of out-of-range samples were compared between the groups with Fisher’s exact test. Third, the covariate-adjusted models were refitted as censored-normal (Tobit) regressions of log-transformed concentrations, with right-censoring at the upper limit for MaR1 and MaR2 and left-censoring at the lower limit for adipolin. In addition, because atopy among controls had been excluded by history only, the primary comparisons were repeated after exclusion of controls with total IgE >100 IU/mL, the upper reference limit of our laboratory. Finally, the adipolin–MaR1 correlation was re-examined separately in samples with adipolin concentrations below and within the calibration range. The post hoc sensitivity analyses and the revised figures were produced with Python 3.11 (SciPy 1.17, statsmodels 0.15, and Matplotlib 3.10).

## 3. Results

### 3.1. Study Population

Eighty-eight participants were analysed: 44 patients with AD and 44 healthy controls. The groups did not differ significantly with respect to age (*p* = 0.055), BMI (*p* = 0.608), or sex distribution (*p* = 0.080). Current smoking was significantly more frequent among controls than among patients (47.7% vs. 18.2%; *p* = 0.003). Demographic, haematological, and biochemical characteristics of both groups are presented in Table 1.

In the patient group, the median disease duration was 84.0 months (interquartile range [IQR] 24.75–180.50). Mean SCORAD was 45.02 ± 9.13 (range 28.0–68.7) and median EASI was 6.30 (3.50–11.62; range 1.2–31.5). By SCORAD, no patient had mild disease, 30 (68.2%) had moderate, and 14 (31.8%) had severe disease, whereas by EASI 23 patients (52.3%) had mild, 19 (43.2%) moderate, and 2 (4.5%) severe disease; the two indices assigned the same severity category in 14 patients (31.8%). In accordance with the eligibility criteria, no patient was receiving systemic therapy, topical therapy, or oral antihistamines for AD at the time of blood sampling.

### 3.2. Haematological and Biochemical Parameters

Neither the systemic inflammatory markers (C-reactive protein, erythrocyte sedimentation rate) nor total IgE differed significantly between the two groups (all *p* > 0.05; Table 1). Among haematological indices, haemoglobin and mean corpuscular haemoglobin concentration were significantly higher in controls (*p* = 0.021 and *p* = 0.049, respectively), while all remaining indices were comparable. Among biochemical parameters, serum LDH (*p* < 0.001), urea (*p* = 0.019), thyroid-stimulating hormone (*p* = 0.043), and high-density lipoprotein cholesterol (*p* = 0.017) were higher in patients than in controls.

Because 41 comparisons were performed within this exploratory laboratory panel (Table 1 and Table S1), the Benjamini–Hochberg procedure was applied. Only the between-group difference in LDH remained significant after correction for multiple testing (q = 0.004); the differences in high-density lipoprotein cholesterol, urea, haemoglobin, thyroid-stimulating hormone, and mean corpuscular haemoglobin concentration did not (q = 0.215–0.335). LDH was therefore examined further in post hoc analyses (Section 3.6).

### 3.3. Serum Adipolin, MaR1, and MaR2

None of the three pre-specified primary outcomes differed significantly between patients and controls (Table 1). Median serum adipolin was 3.16 ng/mL (2.06–11.05) in patients versus 3.23 ng/mL (1.25–12.67) in controls (*p* = 0.211); MaR1 was 140.31 ng/L (94.02–230.17) versus 141.09 ng/L (99.10–257.30) (*p* = 0.815); and MaR2 was 67.56 pg/mL (56.04–79.27) versus 72.29 pg/mL (56.90–87.93) (*p* = 0.512). All three analytes showed markedly right-skewed distributions with wide interquartile ranges in both groups. The corresponding rank-biserial effect sizes were small in magnitude (|r| ≤ 0.16).

### 3.4. Sensitivity Analyses

Given the marked between-group difference in smoking status and the potential for demographic or anthropometric confounding, additional multivariable sensitivity analyses were performed with adjustment for age, sex, BMI, and current smoking status. Using log(1+x)-transformed biomarker concentrations, the adjusted group effect remained non-significant for adipolin (β = 0.210, 95% CI −0.309 to 0.729; *p* = 0.424), MaR1 (β = −0.023, 95% CI −0.263 to 0.217; *p* = 0.849), and MaR2 (β = −0.075, 95% CI −0.221 to 0.070; *p* = 0.306). Model explanatory power was low (adjusted R^2^ = −0.037, 0.009, and 0.030, respectively), and the overall model tests were not significant (all *p* > 0.18). Thus, the absence of a between-group difference for the three primary biomarkers was robust to adjustment for these covariates.

A proportion of measurements lay outside the calibration ranges of the assays (Table S2 and Figure S1). For adipolin, no value exceeded the effective upper calibration limit (112.5 ng/mL), but 31 of 44 patients (70.5%) and 28 of 44 controls (63.6%) had concentrations below the effective lower calibration limit of 6.25 ng/mL (*p* = 0.651), including 12 patients and 19 controls below the dilution-corrected analytical sensitivity (2.15 ng/mL). MaR1 exceeded its upper calibration limit (300 ng/L) in 7 patients (15.9%) and 6 controls (13.6%) (*p* = 1.000; maximum 332.4 ng/L), and MaR2 exceeded its upper limit (100 pg/mL) in 4 patients (9.1%) and 8 controls (18.2%) (*p* = 0.352; maximum 159.8 pg/mL); no MaR1 or MaR2 value fell below the lower limit. When out-of-range values were set to the respective limit, the between-group comparisons remained non-significant (adipolin, *p* = 0.791; MaR1, *p* = 0.744; MaR2, *p* = 0.465), none of the biomarkers correlated with SCORAD or EASI (all *p* ≥ 0.439), and the AUCs were 0.486, 0.480, and 0.455, respectively. The group effect also remained non-significant in the covariate-adjusted Tobit models (adipolin: β = −0.063, 95% CI −0.993 to 0.866, *p* = 0.894; MaR1: β = −0.033, 95% CI −0.294 to 0.227, *p* = 0.802; MaR2: β = −0.087, 95% CI −0.220 to 0.045, *p* = 0.197).

Total IgE exceeded 100 IU/mL in 18 of 44 controls (40.9%) and in 15 of 44 patients (34.1%). After exclusion of these 18 controls, the comparisons between the 44 patients and the remaining 26 controls were again non-significant for adipolin (3.16 vs. 7.73 ng/mL; *p* = 0.470), MaR1 (140.31 vs. 213.39 ng/L; *p* = 0.077), and MaR2 (67.56 vs. 69.01 pg/mL; *p* = 0.947).

### 3.5. Correlation Analyses

Serum adipolin was not correlated with BMI in the pooled cohort (Spearman ρ = −0.098; *p* = 0.363).

**Figure 1:**
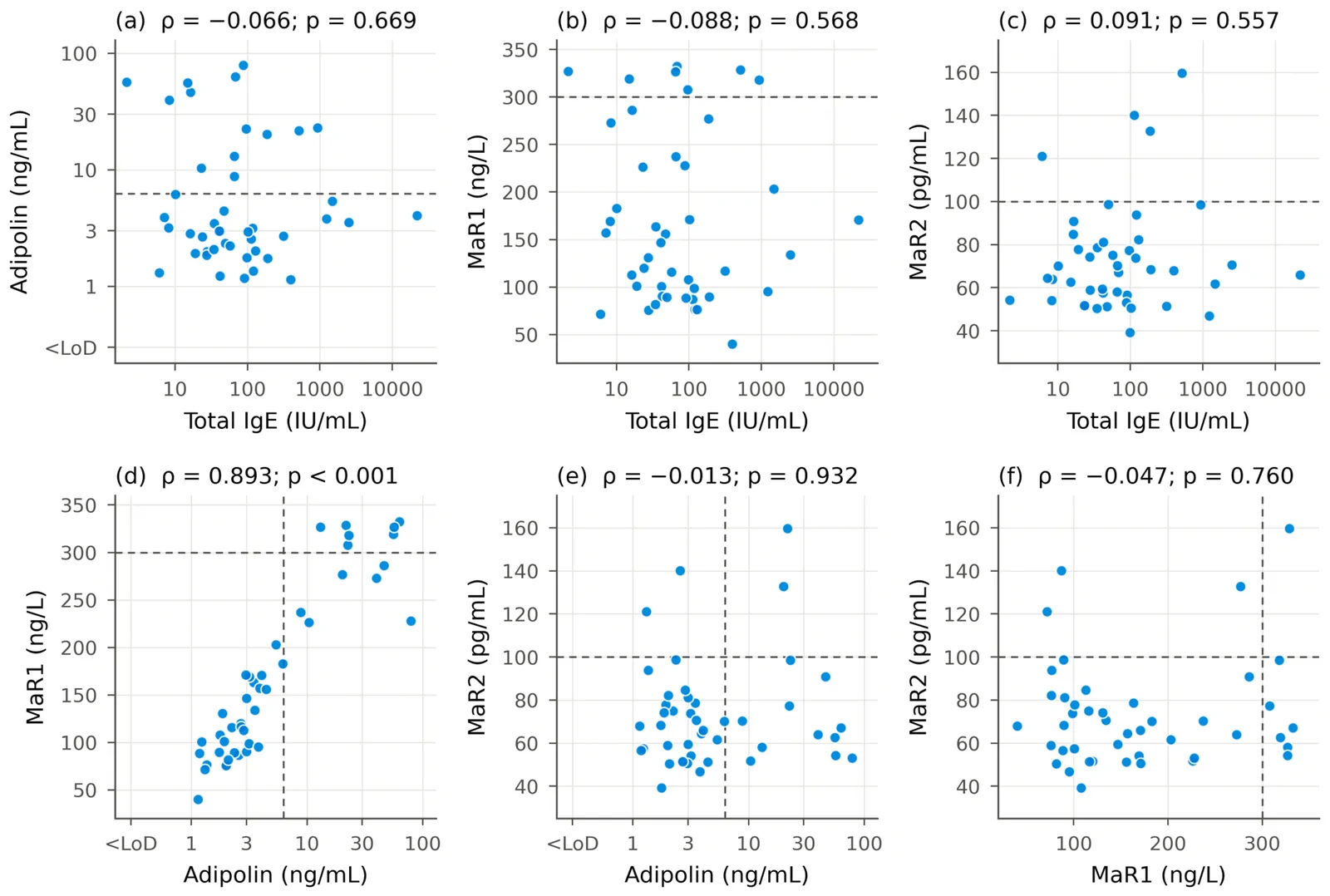
Pairwise correlations between total IgE, adipolin, MaR1, and MaR2 in patients with atopic dermatitis (*n* = 44): (**a**) total IgE and adipolin; (**b**) total IgE and MaR1; (**c**) total IgE and MaR2; (**d**) adipolin and MaR1; (**e**) adipolin and MaR2; (**f**) MaR1 and MaR2. Each panel shows the Spearman coefficient and *p*-value. Each dot represents one patient. Total IgE and adipolin are plotted on logarithmic axes. Dashed lines mark the calibration limits of the assays (adipolin, effective lower limit 6.25 ng/mL; MaR1, upper limit 300 ng/L; MaR2, upper limit 100 pg/mL). <LoD, reported by the laboratory as below the limit of detection.

**Figure 2:**
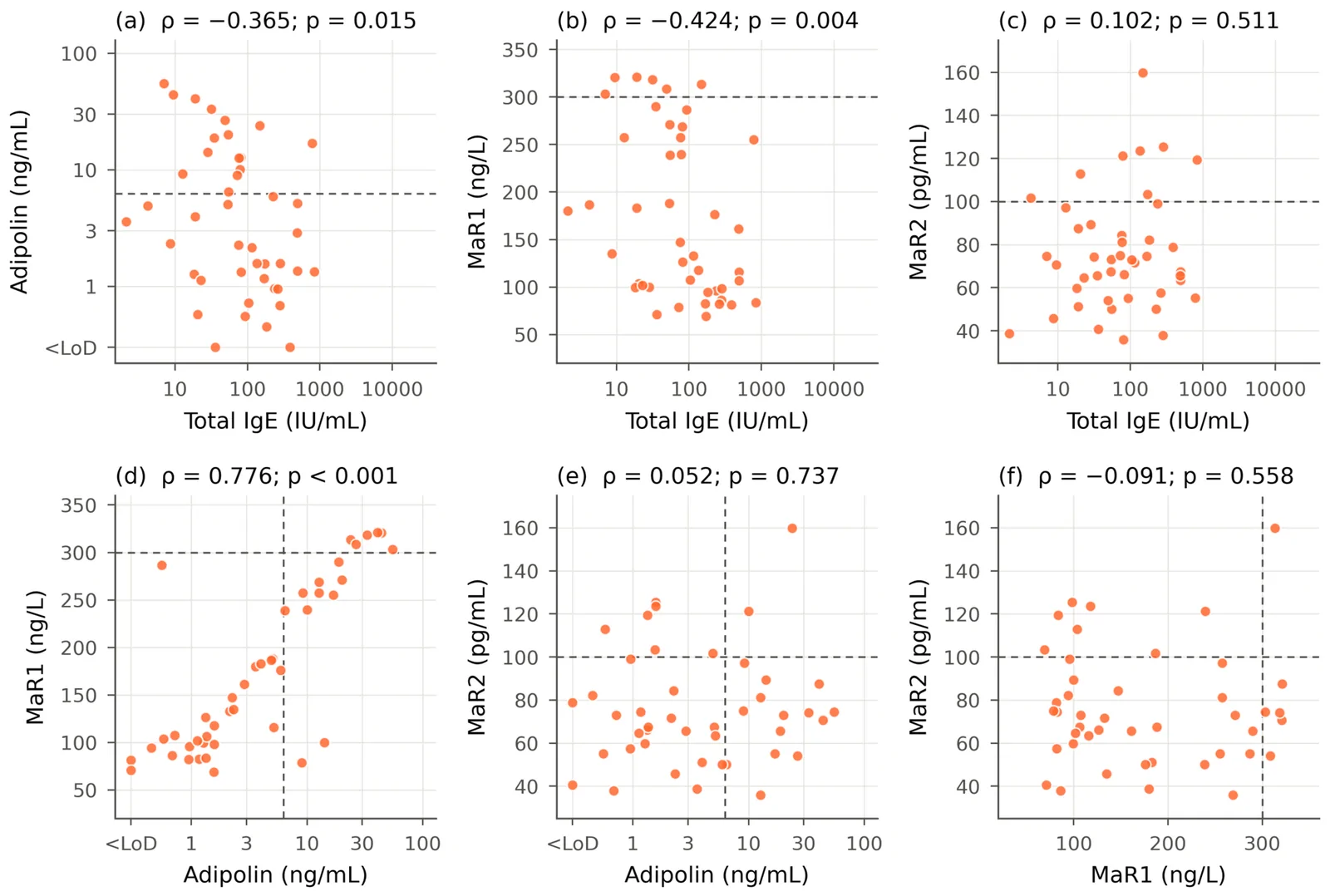
Pairwise correlations between total IgE, adipolin, MaR1, and MaR2 in healthy controls (*n* = 44): (**a**) total IgE and adipolin; (**b**) total IgE and MaR1; (**c**) total IgE and MaR2; (**d**) adipolin and MaR1; (**e**) adipolin and MaR2; (**f**) MaR1 and MaR2. Panel set and layout as in Figure 1. Each dot represents one control participant; dashed lines mark the calibration limits of the assays, as in Figure 1. <LoD, reported by the laboratory as below the limit of detection.

Correlations between total IgE, disease severity indices, and the three biomarkers are summarised in Table 2 (Panel A), and the corresponding scatter plots are shown in Figure 1 (patients) and Figure 2 (controls). In the pooled cohort, total IgE was weakly and inversely correlated with adipolin (ρ = −0.240; *p* = 0.024) and with MaR1 (ρ = −0.239; *p* = 0.025), but not with MaR2 (*p* = 0.322). Group-stratified analysis indicated that both associations were driven entirely by the control group (adipolin: ρ = −0.365, *p* = 0.015; MaR1: ρ = −0.424, *p* = 0.004) and were absent among patients (*p* = 0.669 and *p* = 0.568, respectively).

Within the patient group, neither SCORAD nor EASI correlated with any of the three biomarkers (all *p* ≥ 0.389), whereas the two severity indices were moderately correlated with each other (ρ = 0.467; *p* = 0.001) (Table 2, Panel B).

A strong positive correlation was observed between serum adipolin and MaR1 in the pooled cohort (ρ = 0.815; *p* < 0.001), and this association was reproduced with comparable magnitude within each group separately (patients: ρ = 0.893, *p* < 0.001, Figure 1; controls: ρ = 0.776, *p* < 0.001, Figure 2). In contrast, MaR1 and MaR2 were not correlated with each other (pooled ρ = −0.070; *p* = 0.516), and adipolin was not correlated with MaR2 (pooled ρ = −0.005; *p* = 0.966), in either the pooled cohort or the individual groups.

Because most adipolin concentrations lay below the calibration range, the adipolin–MaR1 association was examined further. The relationship was monotonic but markedly non-linear, with MaR1 rising approximately in proportion to the logarithm of the adipolin concentration and flattening near the upper calibration limit of the MaR1 assay (Figure 1d and Figure 2d): the 23 samples with MaR1 >250 ng/L had a median adipolin concentration of 22.93 ng/mL (17.84–42.35), compared with 2.32 ng/mL (1.33–3.91) in the remaining 65 samples. Conversely, MaR1 exceeded 250 ng/L in 22 of the 29 samples with adipolin within the calibration range but in only 1 of the 59 samples with adipolin below it. The correlation nevertheless persisted among the 59 samples with adipolin below the lower calibration limit (ρ = 0.640; *p* < 0.001), and was ρ = 0.532 (*p* = 0.034) in the 16 samples in which both analytes lay within their calibration ranges. Neither analyte was correlated with triglycerides, total cholesterol, total bilirubin, total protein, or haemoglobin (all |ρ| ≤ 0.16).

### 3.6. Serum Lactate Dehydrogenase, Disease Severity, and Biomarker Levels

**Figure 3:**
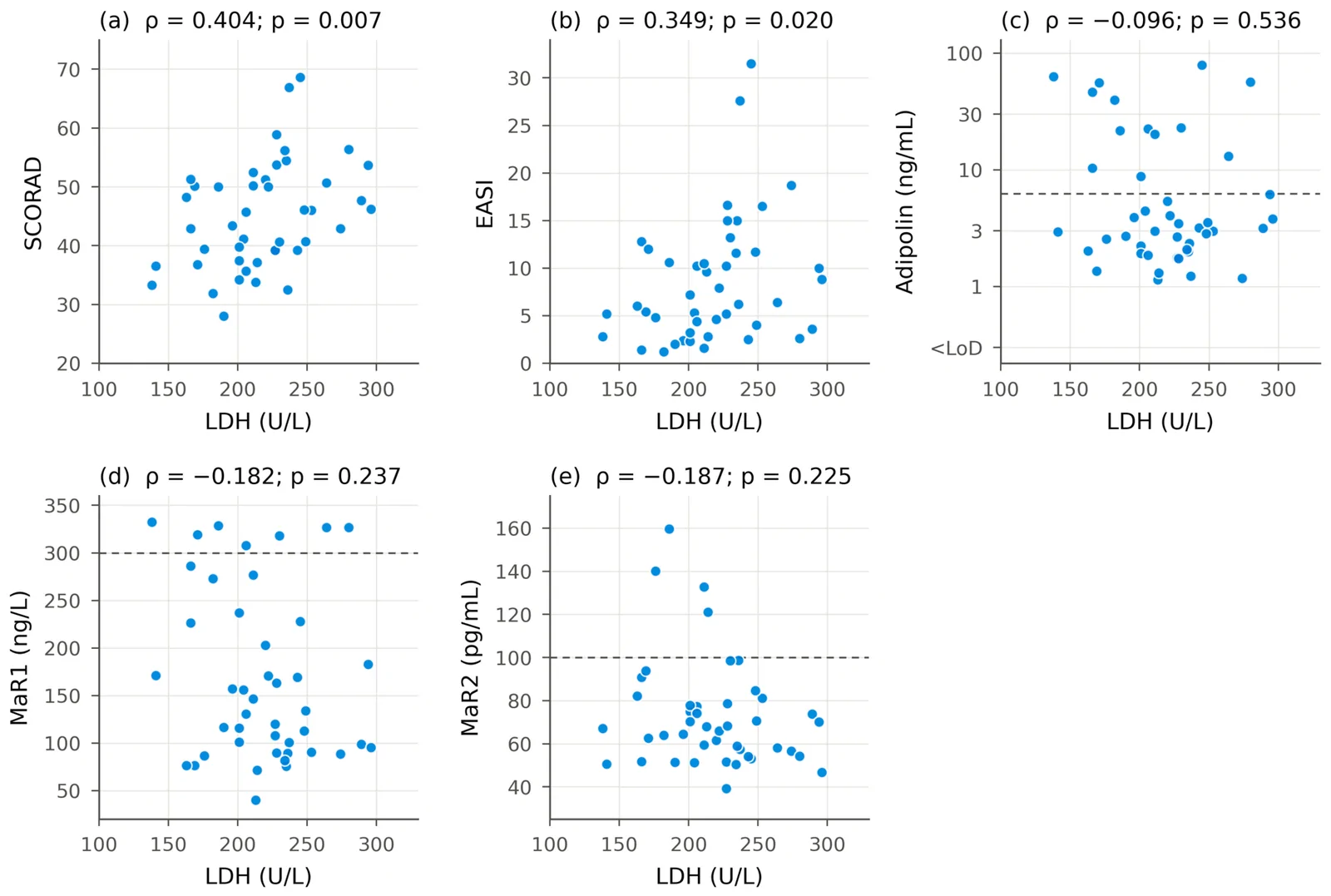
Correlations between serum lactate dehydrogenase and the disease severity indices SCORAD and EASI and the three biomarkers within the patient group (*n* = 44): (**a**) SCORAD; (**b**) EASI; (**c**) adipolin; (**d**) MaR1; (**e**) MaR2. Corresponding coefficients for the pooled cohort and for controls are given in Table 2, Panel C. Axes and dashed lines, as in Figure 1. Each dot represents one patient.

Because LDH was the only laboratory parameter to retain significance after correction for multiple testing, its relationships with disease severity and with the three biomarkers were examined in a post hoc analysis (Table 2, Panel C; Figure 3). Within the patient group, serum LDH was positively correlated with both severity indices, SCORAD (ρ = 0.404; *p* = 0.007) and EASI (ρ = 0.349; *p* = 0.020). These two associations remained significant after Benjamini–Hochberg correction within this family of comparisons (q = 0.033 for both).

In the pooled cohort, LDH showed a weak inverse correlation with MaR1 (ρ = −0.263; *p* = 0.013), whereas no association was detected with adipolin (ρ = −0.018; *p* = 0.869) or MaR2 (ρ = −0.055; *p* = 0.610). Group-stratified analysis did not reproduce the LDH–MaR1 association consistently: the coefficient was of similar direction but not significant among patients (ρ = −0.182; *p* = 0.237) and reached only borderline significance among controls (ρ = −0.311; *p* = 0.040). LDH was not associated with adipolin or MaR2 in either group considered separately (Table 2, Panel C).

### 3.7. Diagnostic Performance

**Figure 4:**
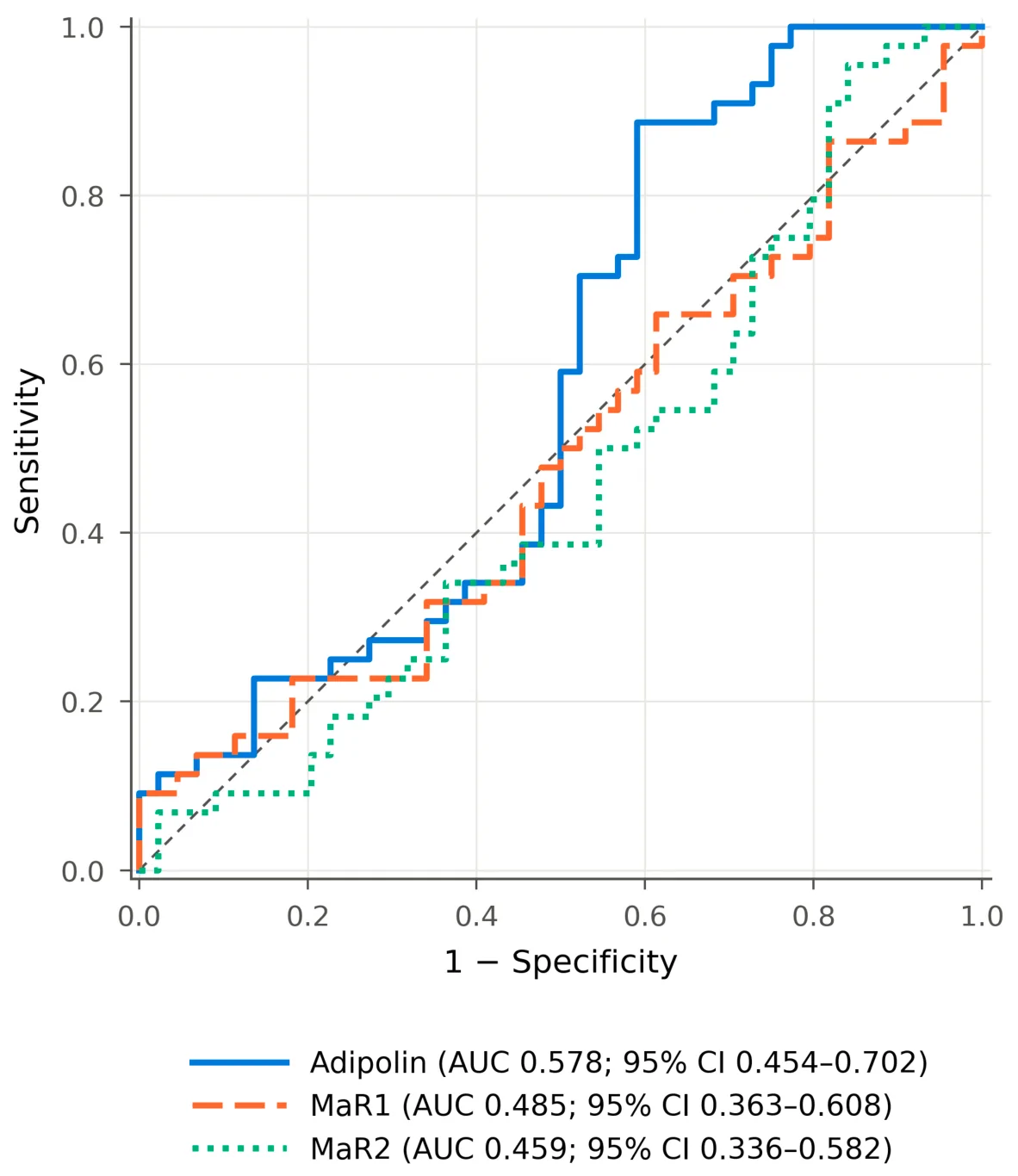
Receiver operating characteristic curves for serum adipolin, MaR1, and MaR2 in discriminating patients with atopic dermatitis from healthy controls. The diagonal dashed line indicates no discrimination. AUC, area under the curve; CI, confidence interval.

Receiver operating characteristic analysis showed that none of the three biomarkers discriminated patients from controls (Table 3 and Figure 4). The area under the curve was 0.578 (95% CI 0.454–0.702; *p* = 0.208) for adipolin, 0.485 (95% CI 0.363–0.608; *p* = 0.816) for MaR1, and 0.459 (95% CI 0.336–0.582; *p* = 0.507) for MaR2. All three confidence intervals included 0.5, that is, no biomarker performed better than chance. Cut-off values, sensitivities, and specificities are therefore not reported, because a threshold derived from a non-discriminating ROC curve has no diagnostic meaning.

## 4. Discussion

This study examined circulating concentrations of MaR1, MaR2, and adipolin/CTRP12 in adults with AD and assessed their associations with clinical disease severity. None of the three primary outcomes differed significantly between patients with AD and healthy controls, and this result persisted after adjustment for age, sex, BMI, and smoking status, with all adjusted confidence intervals including the null value. In addition, none of the biomarkers correlated with SCORAD or EASI within the AD group, and ROC analysis showed no ability to discriminate patients from controls. By contrast, serum LDH was higher in patients with AD and correlated positively with both SCORAD and EASI. Circulating adipolin and MaR1 were strongly correlated, but, as discussed below, this correlation is more likely to be analytical than biological in origin.

These findings should be interpreted in light of the emerging concept that chronic AD may involve not only excessive inflammatory activation but also incomplete resolution of inflammation. SPMs actively terminate inflammation and restore tissue homeostasis. Alterations in omega-3-derived lipid mediators and an imbalance between pro-inflammatory and pro-resolving lipid pathways have been proposed in AD, although direct clinical evidence for individual SPMs remains limited [3,4]. Experimental data indicate that SPMs can modulate several pathways relevant to AD, including type 2 inflammation, IL-17 responses, IgE production, macrophage polarisation, and epidermal barrier function [4]. It was therefore biologically plausible to expect altered circulating maresin concentrations in AD.

To our knowledge, no previous study has compared serum MaR1 concentrations between patients with AD and healthy individuals. In psoriasis, Shaalan et al. reported significantly lower serum MaR1 concentrations than in healthy controls, together with inverse associations with disease duration and the Psoriasis Area and Severity Index (PASI) [26]. Our findings differ: in AD, circulating MaR1 concentrations were neither reduced nor related to clinical severity. This discrepancy is not necessarily a biological contradiction. Psoriasis is primarily characterised by prominent IL-23/IL-17 signalling, whereas AD is dominated by type 2 inflammation, particularly during the acute phase, with additional Th1, Th17, and Th22 components emerging in chronic disease. Pro-resolving mediator profiles may therefore vary with the dominant inflammatory pathway of each dermatosis, and observations in psoriasis cannot be extrapolated directly to AD [4].

Substantial mechanistic evidence supports the involvement of MaR1 in the resolution of inflammation, including in imiquimod-induced skin inflammation, UVB-induced skin injury, and the regulation of epithelial tight-junction proteins [5,6,7,8,13]. The absence of a difference in serum MaR1 in the present study should therefore not be construed as evidence that MaR1-related pathways are irrelevant to AD.

One plausible explanation for the discrepancy between biological rationale and circulating concentrations is the compartmentalisation of lipid mediator activity. SPMs are locally generated, relatively unstable lipid mediators that may act close to their site of synthesis rather than accumulating in the circulation [27]. In psoriasis, lipid mediator profiles differ between skin and blood: certain pro-resolving mediators are detected preferentially in lesional skin, whereas circulating profiles show a distinct pattern, indicating that blood concentrations do not necessarily reflect local lipid mediator biology [28]. A similar dissociation may apply in AD. Serum phospholipid abnormalities observed in AD differ from the characteristic lipid disturbances of the epidermal barrier, further suggesting that systemic and cutaneous lipid metabolism may represent partially distinct biological compartments [29,30]. Consequently, unchanged circulating MaR1 concentrations do not exclude altered MaR1 production, utilisation, degradation, or receptor-mediated activity within lesional or non-lesional AD skin.

The MaR2 findings require more cautious interpretation because the evidence base is considerably smaller than for MaR1. Like MaR1, MaR2 is a DHA-derived macrophage product that inhibits neutrophil recruitment and enhances macrophage phagocytosis at very low concentrations [9], and it attenuated NLRP3 inflammasome activation, Th2 responses, and oxidative stress in a murine model of allergic airway inflammation [10]. However, most of the evidence comes from experimental models, and human clinical data are very sparse [8,13]. Our finding that serum MaR2 concentrations neither differed between patients and controls nor correlated with SCORAD or EASI should therefore be regarded as an initial clinical observation rather than as definitive evidence against a pathogenic or regulatory role for MaR2.

Adipolin/CTRP12 represents a mechanistically distinct but potentially complementary anti-inflammatory pathway. Experimentally, CTRP12 inhibits macrophage production of TNF-α, IL-6, and MCP-1, partly via TGF-βRII/Smad2 signalling [18], and promotes an M2-like macrophage phenotype through the miR-155-5p/LXRα pathway [19]. Clinically, circulating CTRP12 concentrations are reduced in coronary artery disease and correlate inversely with inflammatory cytokines and insulin resistance [20]. To our knowledge, circulating CTRP12 has not previously been studied in AD. Our findings suggest that the systemic reduction in CTRP12 reported in coronary artery disease cannot be extrapolated to chronic cutaneous inflammation.

Adipolin was not associated with BMI in our cohort. BMI was similar in the two groups, and adjustment for BMI, age, sex, and smoking did not materially change the relationship between AD status and circulating adipolin. The null finding for adipolin is therefore unlikely to be explained by differences in adiposity. More generally, the consistency of all three biomarker results after multivariable adjustment indicates that the absence of a between-group difference was not attributable to the measured demographic and metabolic covariates.

The imbalance in smoking between the groups deserves attention: current smoking was considerably more frequent among controls than among patients. This matters because smoking has been linked to altered SPM concentrations. In a conference abstract reporting on 2367 patients with pulmonary arterial hypertension, ever-smoking was associated with lower plasma concentrations of MaR1 and MaR2 measured by targeted liquid chromatography–mass spectrometry [31]. Lower resolvin D1 and resolvin D2 concentrations in sinonasal tissue and lower plasma resolvin E1 concentrations have also been reported in smokers [32,33], although the direction of the smoking effect is not uniform across mediators [31]. These observations derive largely from disease cohorts, from plasma or tissue rather than serum, and in part from ever- rather than current smoking, so their transferability to the present comparison is uncertain. The higher prevalence of smoking among controls could, therefore, in theory, have lowered maresin concentrations in that group and masked a genuine AD-associated reduction. However, after adjustment for smoking together with age, sex, and BMI, no significant group effect was observed for MaR1 or MaR2. This sensitivity analysis reduces, but does not eliminate, the possibility that the smoking imbalance accounts for the null findings.

Serum adipolin and MaR1 were strongly correlated in the pooled cohort and within each group. A biological explanation can be constructed, because both systems converge on resolution-related macrophage functions: CTRP12 suppresses macrophage inflammatory cytokine production and promotes anti-inflammatory polarisation, whereas maresins enhance phagocytosis and efferocytosis [18,19,21]. However, there is no evidence that CTRP12 influences MaR1 biosynthesis, receptor signalling, or metabolism [21], and several features of our data argue against a biological interpretation. First, the correlation is between a protein and a small lipid measured with immunoassays from different manufacturers, whereas MaR1 and MaR2, which derive from the same DHA pathway, were not correlated at all. Second, the two assays ranked the samples almost identically across the whole concentration range, and essentially the same subset of samples read high in both, with MaR1 exceeding 250 ng/L in 22 of the 29 samples with quantifiable adipolin but in only 1 of the 59 samples with adipolin below the calibration range. Third, the correlation persisted among samples in which adipolin lay below the lower calibration limit, a range in which the assay cannot be assumed to quantify the analyte. Fourth, adipolin and MaR1 showed virtually identical correlations with total IgE. Taken together, these observations suggest that the two assays partly captured a shared non-biological signal rather than coordinated regulation of two mediators. Candidate explanations are non-specific binding of sample components, a recognised source of background signal in ELISA [34], or another matrix effect in a subset of sera, or a positional effect if samples were loaded in the same order on the plates of both assays. We could not distinguish between these possibilities, because individual duplicate readings, plate layouts, and spike-recovery or dilution-linearity data were not available; neither analyte was related to serum lipids, bilirubin, total protein, or haemoglobin. Because patient and control samples were distributed across the same plates, an effect of this kind would not be expected to bias the between-group comparisons. We therefore regard the adipolin–MaR1 correlation as a probable analytical artefact until it is reproduced with an orthogonal method, and we draw no biological inference from it.

MaR2 was not correlated with either adipolin or MaR1. If the adipolin–MaR1 correlation is analytical in origin, the absence of a MaR1–MaR2 correlation carries little biological information, and we do not interpret it as evidence that the two maresins are regulated differently. Human data on circulating MaR2 remain very limited.

None of the three primary biomarkers correlated with SCORAD or EASI, suggesting that they are not suitable as circulating severity markers, at least within the clinical spectrum of our cohort. They were also not associated with total IgE in the AD group. Other SPMs, particularly resolvin D1 and 17-hydroxydocosahexaenoic acid, inhibit human B-cell IgE production and IL-4/STAT6-dependent IgE class switching [35]. However, these mechanisms cannot be assumed to apply to circulating MaR1 or MaR2. Furthermore, total IgE does not consistently reflect clinical disease severity and captures only one aspect of the complex immunological heterogeneity of allergic disease [36]. The absence of an association between maresins or adipolin and total IgE therefore does not necessarily contradict their proposed immunoregulatory functions.

The two severity indices gave discordant pictures of the cohort: by SCORAD all patients had moderate or severe disease, whereas by EASI about half had mild disease, and the indices were only moderately correlated (ρ = 0.467). SCORAD and EASI differ in construction. SCORAD assigns up to 20 of its 103 points to patient-reported pruritus and sleep loss and weights lesion intensity heavily, whereas EASI is driven by the extent of involvement in four body regions and has no subjective component [37]; such structural differences are known to affect severity classification [38]. In addition, our patients were untreated at sampling, having used no topical therapy or emollients for at least two weeks and no oral antihistamines, which may have increased pruritus, sleep loss, and xerosis, all of which raise SCORAD but not EASI. Because the objective and subjective components of SCORAD were not recorded separately, this explanation could not be verified, and misclassification of severity by either index cannot be excluded. Nevertheless, the absence of any correlation between the three biomarkers and either index makes it unlikely that the choice of severity instrument explains the null severity findings.

Median total IgE was numerically higher in controls than in patients, and 18 of the 44 controls (40.9%) had values above the laboratory reference limit of 100 IU/mL. Controls were screened for atopic disease by history only; specific IgE measurement and skin prick testing were not performed, so subclinical atopic sensitisation among controls cannot be excluded. When these controls were excluded, the comparisons remained non-significant, although MaR1 tended to be lower in patients than in the remaining 26 controls (*p* = 0.077). This post hoc, unadjusted comparison should be regarded as exploratory, but it indicates that a difference in MaR1 between patients with AD and strictly non-atopic controls cannot be excluded.

LDH provided an informative contrast. Unlike the three pre-specified biomarkers, serum LDH was significantly higher in patients with AD and correlated positively with both SCORAD and EASI. Previous studies have consistently reported increased LDH concentrations in AD and their associations with disease severity [39,40,41]. LDH may reflect tissue injury and epidermal barrier disturbance rather than a single immunological pathway; in one cohort, higher LDH was accompanied by greater transepidermal water loss in patients colonised with *Staphylococcus aureus* [42]. The LDH findings therefore provide useful internal context: MaR1, MaR2, and adipolin did not differ in a cohort in which an established laboratory marker of AD activity was detectably altered. This supports the interpretation that the null primary findings are specific to these biomarkers and do not reflect an absence of measurable disease-related biological variation.

Similarly, ROC analysis showed that none of the three primary biomarkers discriminated patients with AD from healthy controls, in keeping with the largely overlapping distributions and small unadjusted effect sizes. Although high diagnostic performance has been reported for serum MaR1 in psoriasis [26], the present findings indicate that this cannot be generalised across diseases. Circulating MaR1, MaR2, and adipolin therefore do not appear useful as standalone diagnostic biomarkers for AD in the population studied.

Several limitations warrant consideration. Firstly, the sample consisted of 44 patients and 44 controls, which restricts the precision of detecting small effects and conducting subgroup analyses. Although the unadjusted effects observed were minimal and remained non-significant following multivariable adjustment, the study does not establish statistical equivalence between the groups. Secondly, because the biomarkers were measured at a single time point in prevalent cases, the design permits the assessment of associations but not of temporal or causal relationships; longitudinal changes during exacerbation, remission, or treatment may differ from those observed at a single time point. Thirdly, there were differences in age and, more notably, smoking status between the patient and control groups. While the main findings persisted after adjusting for these variables, residual confounding cannot be entirely ruled out. Sex-disaggregated analyses were not performed because the sample size did not provide adequate statistical power for subgroup comparisons; sex was instead included as a covariate in the multivariable models. Fourthly, the control group was defined by history; 40.9% of controls had total IgE above the laboratory reference limit, and specific IgE measurement and skin prick testing were not performed. Fifthly, patients were required to be free of systemic and topical treatment, emollients, and oral antihistamines, which limits generalisability to treated populations, and the components of SCORAD were not recorded separately, so the discordance between SCORAD and EASI could not be explored.

The most important limitations of this study are analytical. With the 1:5 pre-assay dilution, 67.0% of adipolin concentrations fell below the lowest calibrator and 35.2% below the dilution-corrected analytical sensitivity, so adipolin was not reliably quantified in most samples. The null adipolin findings therefore indicate only that no between-group difference was detectable with this assay, and re-assay at a lower dilution would be required for a definitive comparison. For MaR1 and MaR2, 14.8% and 13.6% of values, respectively, exceeded the highest calibrator and were extrapolated rather than re-assayed after dilution, although the excess was small for MaR1 (at most 11% above the limit) and all conclusions were unchanged when out-of-range values were censored. Intra- and inter-assay precision could not be determined from our own data, and in-house verification of dilution linearity and recovery, which is recommended for commercial kits [34], was not available to us; the adipolin–MaR1 correlation discussed above suggests that part of the measured signal may be non-specific. More generally, analytical considerations are crucial for the study of maresins. Although ELISA-based measurements have been employed in numerous clinical studies, the absolute concentrations of MaR1 exhibit significant variability across published cohorts, indicating considerable inter-assay and pre-analytical heterogeneity. For example, with kits from other manufacturers, synovial fluid MaR1 concentrations were in the ng/mL range and MaR1 and MaR2 were moderately correlated (ρ = 0.620) [43], whereas the two maresins were uncorrelated in our serum samples. Additionally, SPMs include structurally similar positional and stereochemical isomers, which pose challenges for differentiation using antibody-based assays [44,45]. Comparative analyses reveal that ELISA and liquid chromatography–tandem mass spectrometry (LC-MS/MS) may significantly differ in absolute SPM quantification, though ELISA remains useful for evaluating relative differences within studies [44,45]. These methodological issues are particularly pertinent for MaR2, as validated human serum data are notably limited. Consequently, future research incorporating mass-spectrometry-based lipid mediator profiling would offer more robust analytical validation.

## 5. Conclusions

In summary, circulating concentrations of MaR1, MaR2, and adipolin/CTRP12 did not differ significantly between adults with AD and healthy controls. This finding persisted after adjustment for age, sex, BMI, and smoking status and in sensitivity analyses that accounted for measurements outside the assay calibration ranges and for controls with raised total IgE. None of these three biomarkers showed an association with SCORAD or EASI, nor did they provide clinically meaningful diagnostic discrimination. Consequently, their use as standalone circulating diagnostic or severity biomarkers for AD is not supported. Nevertheless, this does not preclude the potential local involvement of maresin- or CTRP12-related pathways within the skin, especially considering the compartmentalised nature of lipid mediator biology. These conclusions are qualified by analytical limitations: most adipolin concentrations lay below the calibration range of the assay, a minority of maresin values lay above it, and the strong adipolin–MaR1 correlation showed features of an analytical artefact and should not be interpreted biologically. Conversely, the elevation of LDH and its association with clinical severity scores align with existing AD literature. Larger longitudinal studies that include strictly non-atopic controls and integrate paired blood and skin sampling with analytically validated assays and mass-spectrometry-based lipid mediator profiling may more accurately delineate the relevance of inflammation-resolution pathways in AD.

## Figures and Tables

**Table 1:** Demographic, laboratory, and biomarker characteristics of patients with atopic dermatitis and healthy controls.

Variable	Unit	AD Patients (*n* = 44)	Controls (*n* = 44)	*p*
Age	years	31.50 (24.00–43.00)	25.00 (23.00–36.00)	0.055
Female sex	n (%)	31 (70.5)	23 (52.3)	0.080
Body mass index	kg/m^2^	25.60 ± 2.93	26.02 ± 4.51	0.608
Current smoking	n (%)	8 (18.2)	21 (47.7)	**0.003**
C-reactive protein	mg/L	3.11 (3.11–3.52)	3.11 (3.11–3.70)	0.738
Erythrocyte sedimentation rate	mm/h	10.50 (5.75–18.25)	9.00 (5.00–16.25)	0.482
Total IgE	IU/mL	61.45 (23.70–123.00)	77.65 (27.07–193.82)	0.634
HbA1c	%	5.33 ± 0.29	5.44 ± 0.31	0.071
White blood cells	×10^9^/L	7.24 (6.35–8.60)	7.28 (6.53–8.70)	0.593
Haemoglobin	g/dL	14.35 (13.65–15.43)	15.30 (14.28–16.40)	**0.021**
Haematocrit	%	43.69 ± 3.86	44.96 ± 4.32	0.149
Mean corpuscular volume	fL	85.85 (82.77–87.88)	86.45 (82.83–89.93)	0.481
Mean corpuscular Hb concentration	g/dL	32.94 ± 1.53	33.51 ± 1.16	**0.049**
Red cell distribution width	%	13.00 (12.17–13.70)	12.90 (12.47–13.62)	0.933
Platelets	×10^9^/L	287.91 ± 73.96	268.84 ± 56.02	0.177
Mean platelet volume	fL	10.31 ± 0.99	10.25 ± 0.71	0.712
Lymphocytes	×10^9^/L	2.22 ± 0.65	2.35 ± 0.57	0.319
Neutrophils	×10^9^/L	4.00 (3.50–5.10)	4.21 (3.56–5.64)	0.448
Monocytes	×10^9^/L	0.55 (0.45–0.71)	0.58 (0.49–0.73)	0.193
Eosinophils	×10^9^/L	0.20 (0.11–0.31)	0.15 (0.09–0.22)	0.065
Glucose	mg/dL	87.50 ± 9.75	88.55 ± 7.63	0.577
Total cholesterol	mg/dL	183.75 ± 33.38	171.92 ± 40.19	0.137
HDL cholesterol	mg/dL	48.66 ± 10.16	43.01 ± 11.63	**0.017**
LDL cholesterol	mg/dL	102.15 (90.68–128.90)	101.00 (75.75–114.25)	0.120
Triglycerides	mg/dL	103.00 (77.50–179.00)	111.50 (67.85–176.30)	0.907
Aspartate aminotransferase	U/L	20.00 (16.75–23.00)	19.00 (16.00–22.25)	0.710
Alanine aminotransferase	U/L	16.00 (11.75–21.00)	17.00 (11.00–30.00)	0.738
Lactate dehydrogenase	U/L	217.52 ± 38.70	183.39 ± 39.62	**<0.001**
Total protein	g/dL	7.58 ± 0.59	7.45 ± 0.53	0.296
Albumin	g/dL	4.70 (4.47–4.93)	4.80 (4.60–4.93)	0.296
Urea	mg/dL	28.50 ± 7.56	24.93 ± 6.37	**0.019**
Creatinine	mg/dL	0.76 (0.71–0.92)	0.80 (0.70–0.89)	0.897
Uric acid	mg/dL	5.05 (4.10–6.25)	4.90 (4.18–5.55)	0.783
Thyroid-stimulating hormone	mIU/L	2.01 (1.40–2.57)	1.66 (1.10–2.15)	**0.043**
**Adipolin**	ng/mL	3.16 (2.06–11.05)	3.23 (1.25–12.67)	0.211
**MaR1**	ng/L	140.31 (94.02–230.17)	141.09 (99.10–257.30)	0.815
**MaR2**	pg/mL	67.56 (56.04–79.27)	72.29 (56.90–87.93)	0.512

Data are presented as median (25th–75th percentile), mean ± standard deviation, or n (%). Categorical variables were compared with Pearson’s chi-square test; continuous variables with the independent-samples *t*-test or the Mann–Whitney U test according to distribution. AD, atopic dermatitis; Hb, haemoglobin; HbA1c, glycated haemoglobin; HDL, high-density lipoprotein; IgE, immunoglobulin E; LDL, low-density lipoprotein; MaR1, maresin-1; MaR2, maresin-2. Bold indicates *p* < 0.05 (unadjusted for multiple comparisons).

**Table 2:** Spearman rank correlations.

***Panel A. Whole cohort and group-stratified analyses***					
**Variable Pair**	**Whole Cohort (*n* = 88)**		**AD Patients (*n* = 44)**		**Controls (*n* = 44)**
Total IgE–Adipolin	−0.240 (0.024)		−0.066 (0.669)		−0.365 (0.015)
Total IgE–MaR1	−0.239 (0.025)		−0.088 (0.568)		−0.424 (0.004)
Total IgE–MaR2	0.107 (0.322)		0.091 (0.557)		0.102 (0.511)
Adipolin–MaR1	0.815 (<0.001)		0.893 (<0.001)		0.776 (<0.001)
Adipolin–MaR2	−0.005 (0.966)		−0.013 (0.932)		0.052 (0.737)
MaR1–MaR2	−0.070 (0.516)		−0.047 (0.760)		−0.091 (0.558)
***Panel B. Disease severity indices, patients only (n = 44)***					
**Variable Pair**		**ρ**		***p***	
SCORAD–EASI		0.467		0.001	
Total IgE–SCORAD		0.026		0.865	
Total IgE–EASI		0.209		0.172	
SCORAD–Adipolin		0.098		0.529	
SCORAD–MaR1		0.023		0.884	
SCORAD–MaR2		−0.099		0.521	
EASI–Adipolin		−0.116		0.452	
EASI–MaR1		−0.133		0.389	
EASI–MaR2		−0.019		0.901	
***Panel C. Serum lactate dehydrogenase (*** **post hoc *analysis)***					
**Variable Pair**	**Whole Cohort (*n* = 88)**		**AD Patients (*n* = 44)**		**Controls (*n* = 44)**
LDH–SCORAD	—		0.404 (0.007)		—
LDH–EASI	—		0.349 (0.020)		—
LDH–Adipolin	−0.018 (0.869)		−0.096 (0.536)		−0.036 (0.818)
LDH–MaR1	−0.263 (0.013)		−0.182 (0.237)		−0.311 (0.040)
LDH–MaR2	−0.055 (0.610)		−0.187 (0.225)		0.045 (0.771)

Values are Spearman’s ρ with the corresponding *p*-value in parentheses (Panels A and C). Correlations involving SCORAD or EASI are computed within the patient group only and are therefore not applicable (—) to the pooled cohort or the control group. AD, atopic dermatitis; EASI, Eczema Area and Severity Index; IgE, immunoglobulin E; LDH, lactate dehydrogenase; MaR1, maresin-1; MaR2, maresin-2; SCORAD, Scoring Atopic Dermatitis. Except where stated in the text, *p*-values are unadjusted for multiple comparisons.

**Table 3:** Diagnostic performance of serum adipolin, MaR1, and MaR2 for discriminating patients with atopic dermatitis from healthy controls.

Biomarker	AUC	95% CI	*p*
Adipolin	0.578	0.454–0.702	0.208
MaR1	0.485	0.363–0.608	0.816
MaR2	0.459	0.336–0.582	0.507

AUC, area under the curve; CI, confidence interval; MaR1, maresin-1; MaR2, maresin-2. The *p*-value tests the null hypothesis AUC = 0.5. Higher values were tested as indicative of disease in all three analyses.

## Data Availability

The data presented in this study are available on request from the corresponding author due to privacy and ethical restrictions.

## Supplementary Materials

The following supporting information can be downloaded at: https://www.mdpi.com/article/10.3390/medicina62101923/s1, Figure S1: Distribution of serum adipolin, MaR1, and MaR2 concentrations in relation to the assay calibration limits; Table S1: Routine laboratory variables included in the exploratory comparison family but not shown in Table 1; Table S2: Measurements outside the assay calibration ranges and results of the sensitivity analyses.
