# Supplementary material for: Circulating Pro-Resolving Mediators Maresin-1 and Maresin-2 and Adipolin/CTRP12 in Adults with Atopic Dermatitis: A Case–Control Study
Source: Medicina (Kaunas). 2026 Oct 6;62(10):1923. doi: 10.3390/medicina62101923 (PMC13707223; doi:10.3390/medicina62101923)
Supplement: Supplementary file 1 [file medicina-62-01923-s001.zip › medicina-4571031-supplementary.pdf]

Supplementary Materials

Circulating Pro-Resolving Mediators Maresin-1 and Maresin-2 and Adipolin/CTRP12 in Adults with Atopic Dermatitis: A Case–Control Study (medicina-4571031)

Table S1. Routine laboratory variables included in the family of 41 exploratory between-group comparisons but not shown in Table 1.

**Variable**	**Unit**	**AD patients (n = 44)**	**Controls (n = 44)**	**p**	**q**
Red blood cells	$\times 10^{12}$/L	5.12 (4.84–5.36)	5.21 (4.89–5.63)	0.299	0.681
Mean corpuscular haemoglobin	pg	28.55 (26.95–29.40)	29.25 (27.40–30.30)	0.079	0.359
Platelet distribution width	fL	11.75 (10.60–13.90)	11.70 (10.65–12.72)	0.413	0.807
Plateletcrit	%	0.29 ± 0.07	0.27 ± 0.06	0.123	0.460
Basophils	$\times 10^{9}$/L	0.02 (0.01–0.04)	0.02 (0.01–0.03)	0.798	0.963
Immature granulocytes	$\times 10^{9}$/L	0.02 (0.01–0.03)	0.03 (0.02–0.03)	0.403	0.807
Estimated glomerular filtration rate [a]	mL/min/1.73 m^2	90.00 (90.00–90.00)	90.00 (90.00–90.00)	0.955	0.977
VLDL cholesterol	mg/dL	20.60 (15.50–35.80)	23.00 (12.95–34.88)	0.977	0.977
Total bilirubin	mg/dL	0.58 (0.43–0.85)	0.59 (0.41–0.81)	0.884	0.977
Direct bilirubin	mg/dL	0.17 (0.13–0.24)	0.17 (0.12–0.25)	0.834	0.977
Indirect bilirubin	mg/dL	0.42 (0.30–0.59)	0.41 (0.31–0.55)	0.751	0.962

Data are median (25th–75th percentile) or mean ± standard deviation; comparisons by Mann–Whitney U test or independent-samples t-test according to distribution. q, Benjamini–Hochberg-adjusted p value within the family of 41 comparisons (the 30 variables in Table 1 plus the 11 variables shown here). [a] Values above 90 mL/min/1.73 m^2 are reported by the laboratory as 90. AD, atopic dermatitis; VLDL, very-low-density lipoprotein.

Table S2. Measurements outside the assay calibration ranges and results of the sensitivity analyses.

Panel A. Measurements outside the calibration range

Analyte	Calibration range	Below lower limit, n (%): AD / controls	Above upper limit, n (%): AD / controls	p [b]
Adipolin	6.25–112.5 ng/mL [a]	31 (70.5) / 28 (63.6)	0 / 0	0.651
MaR1	0.5–300 ng/L	0 / 0	7 (15.9) / 6 (13.6)	1.000
MaR2	1.56–100 pg/mL	0 / 0	4 (9.1) / 8 (18.2)	0.352

Panel B. Primary analyses repeated with out-of-range values set to the calibration limit

Analyte	Original p (Mann–Whitney U)	Censored p (Mann–Whitney U)	Censored AUC	Original adjusted p (OLS, log(1+x))	Tobit group β (95% CI); p [c]
Adipolin	0.211	0.791	0.486	0.424	−0.063 (−0.993 to 0.866); 0.894
MaR1	0.815	0.744	0.480	0.849	−0.033 (−0.294 to 0.227); 0.802
MaR2	0.512	0.465	0.455	0.306	−0.087 (−0.220 to 0.045); 0.197

Panel C. Primary comparisons after exclusion of controls with total IgE >100 IU/mL

Analyte	AD patients (n = 44)	Controls with IgE ≤100 IU/mL (n = 26)	p
Adipolin (ng/mL)	3.16 (2.06–11.05)	7.73 (2.27–17.66)	0.470
MaR1 (ng/L)	140.31 (94.02–230.17)	213.39 (128.52–282.76)	0.077
MaR2 (pg/mL)	67.56 (56.04–79.27)	69.01 (54.31–83.48)	0.947

[a] Effective range in undiluted serum after the 1:5 pre-assay dilution (kit range 1.25–22.5 ng/mL); 12 patients and 19 controls were below the dilution-corrected analytical sensitivity of 2.15 ng/mL. [b] Fisher's exact test for the between-group difference in the proportion of out-of-range samples. [c] Censored-normal regression of log-transformed concentration on study group, adjusted for age, sex, body mass index, and current smoking; left-censored at 6.25 ng/mL for adipolin and right-censored at 300 ng/L and 100 pg/mL for MaR1 and MaR2. AD, atopic dermatitis; AUC, area under the receiver operating characteristic curve; CI, confidence interval; IgE, immunoglobulin E; MaR1, maresin-1; MaR2, maresin-2; OLS, ordinary least squares.

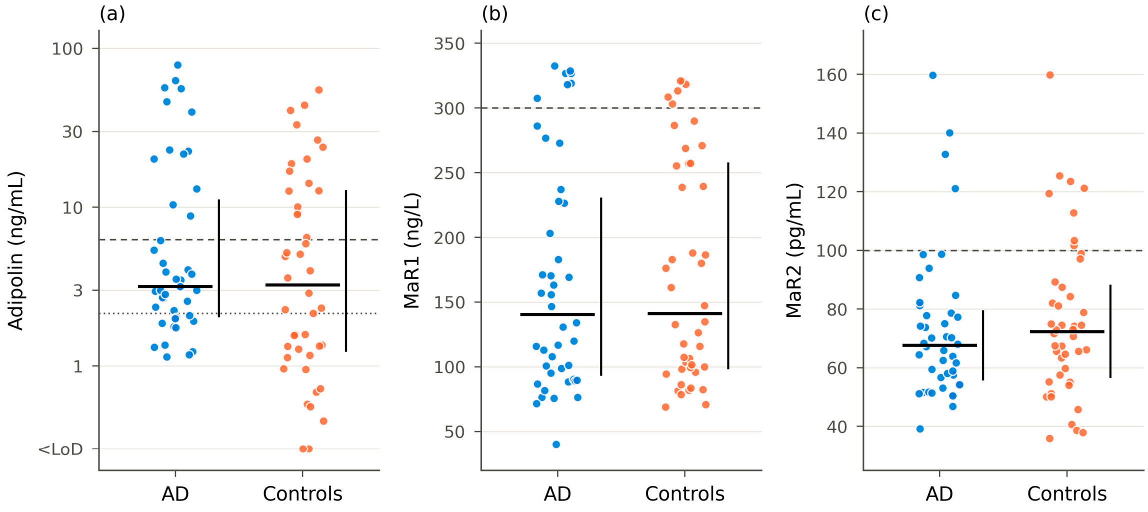

Figure S1. Distribution of serum (a) adipolin, (b) MaR1, and (c) MaR2 concentrations in patients with atopic dermatitis (AD; n = 44) and healthy controls (n = 44) in relation to the assay calibration limits. Each dot is one participant; horizontal bars show medians and vertical bars the 25th–75th percentiles. Dashed lines mark the calibration limits (adipolin: effective lower limit 6.25 ng/mL after the 1:5 pre-assay dilution; MaR1: upper limit 300 ng/L; MaR2: upper limit 100 pg/mL); the dotted line in panel (a) marks the dilution-corrected analytical sensitivity of the adipolin assay (2.15 ng/mL). Adipolin is plotted on a logarithmic axis; <LoD, reported by the laboratory as below the limit of detection.
